# Posterior rat eye during acute intraocular pressure elevation studied using polarization sensitive optical coherence tomography

**DOI:** 10.1364/BOE.8.000298

**Published:** 2016-12-16

**Authors:** Stanislava Fialová, Marco Augustin, Corinna Fischak, Leopold Schmetterer, Stephan Handschuh, Martin Glösmann, Michael Pircher, Christoph K. Hitzenberger, Bernhard Baumann

**Affiliations:** 1 Center for Medical Physics and Biomedical Engineering, Medical University of Vienna, Waehringer Guertel 18-20, 1090 Vienna, Austria; 2 Department of Clinical Pharmacology, General Hospital and Medical University of Vienna, Waehringer Guertel 18-20, 1090 Vienna, Austria; 3 Singapore Eye Research Institute, The Academia, 20 College Road, Discovery Tower Level 6, 169856 Singapore, Republic of Singapore; 4 Lee Kong Chian School of Medicine, Nanyang Technological University, Novena Campus, 11 Mandalay Road, 308232 Singapore, Republic of Singapore; 5 VetCore Facility for Research and Technology, University of Veterinary Medicine Vienna, Veterinaerplatz 1, 1210 Vienna, Austria

**Keywords:** (110.4500) Optical coherence tomography, (130.5440) Polarization-selective devices, (170.3880) Medical and biological imaging, (170.5755) Retina scanning

## Abstract

Polarization sensitive optical coherence tomography (PS-OCT) operating at 840 nm with axial resolution of 3.8 µm in tissue was used for investigating the posterior rat eye during an acute intraocular pressure (IOP) increase experiment. IOP was elevated in the eyes of anesthetized Sprague Dawley rats by cannulation of the anterior chamber. Three dimensional PS-OCT data sets were acquired at IOP levels between 14 mmHg and 105 mmHg. Maps of scleral birefringence, retinal nerve fiber layer (RNFL) retardation and relative RNFL/retina reflectivity were generated in the peripapillary area and quantitatively analyzed. All investigated parameters showed a substantial correlation with IOP. In the low IOP range of 14-45 mmHg only scleral birefringence showed statistically significant correlation. The polarization changes observed in the PS-OCT imaging study presented in this work suggest that birefringence of the sclera may be a promising IOP-related parameter to investigate.

## 1. Introduction

Glaucoma is a group of optic neuropathies characterized by progressive loss of axons and retinal ganglion cells associated with visual field loss. The disease can be divided into several types: open-angle glaucoma, angle-closure glaucoma, normal-tension glaucoma, congenital glaucoma and another variants [1]. Common to all of them is the damage of retinal ganglion cells and the fact that lowering of the intraocular pressure (IOP) slows the progress of the disease [1]. Therefore, there is evidence that IOP is important in this disease, although its contribution to the pathogenesis of glaucoma still requires more investigation [1]. The sclera, as protective shield of the eye, was hypothesized to play a key role in optic nerve head (ONH) damage [2]. At increased IOP, the biomechanical and structural properties of the sclera are altered as was shown in previous studies [3,4]. Biomechanical properties of the sclera and the lamina cribrosa may to a certain degree explain why some patients are more sensitive to increased IOP than others [5]. As such, there is considerable interest to study alterations of the sclera associated with increased IOP and understand their role in the pathogenesis of glaucoma.

Anterior and posterior segment sclera were previously imaged in vivo in humans using polarization sensitive optical coherence tomography (PS-OCT). PS-OCT enables the measurement of optical tissue properties, such as the birefringence caused by collagen fibers in the sclera or by axons in the retinal nerve fiber layer (RNFL) [3,6–9]. While in-vivo clinical studies allow the diagnosis and monitoring of physiological and pathological states, only limited interventions can be done in humans. Hence, to deepen the understanding on the effects of the IOP level on the posterior eye, animal studies are beneficial.

Studies investigating the sclera ex vivo and in animal models were performed by various groups [3,10–16]. Main target of these investigations was the change of structural properties, e.g. collagen organization of the sclera as a response to different stress levels. This allows studying the elasticity and thus the mechanical properties of the sclera. In vivo animal studies were mainly performed during an artificially induced increase of the IOP (e.g. by cannulation of the eye). OCT and its functional extensions hereby enable the determination of various structural and functional parameters in vivo. Retinal perfusion at various IOP levels was studied using OCT-based optical microangiography [17–19]. The ONH and the optic nerve itself were studied under physiological conditions [20] and after IOP elevation using conventional OCT [21,22]. Reflectivity and thickness changes of retinal layers were reported [23,24].

We recently developed a high resolution PS-OCT system for small animal imaging [25]. With our prototype we can assess a threefold image contrast: morphological information based on reflectivity, polarization information resulting from polarization properties of the tissue structure and motion contrast based on the movement of the blood cells in vessels. The system was previously used to investigate healthy pigmented and non-pigmented rodents [25] as well as longitudinal retinal changes in a mutant mouse model of retinal and choroidal neovascularization [26]. In this article, the system was used for investigating the retina and the sclera during experiments with acutely increased IOP in non-pigmented Sprague Dawley rats. Hereby, quantitative parameters were determined and comprise information about changes in the morphology, e.g. deformation of the ONH, together with polarization properties such as scleral birefringence. Since earlier studies [17–19] already investigated the microvascular response to an acute IOP increase, we exclusively focus on changes of tissue morphology and polarization properties in this article. Furthermore, ex vivo micro-computed tomography (µCT) was performed to identify anatomical relations in and around the eye ball.

This work demonstrates the potential of OCT and its functional extensions to study tissue microstructures with intrinsic contrast in preclinical studies through quantitative evaluation of optical tissue properties. The experiment extends the aforementioned in vivo studies addressing IOP elevation and underscores the promising role that PS-OCT might play for further deepening the understanding of the causal relationship of distinct ocular structures in the pathogenesis of ophthalmic diseases.

## 2. Methodology

### 2.1 High resolution polarization sensitive OCT

**Fig. 1:**
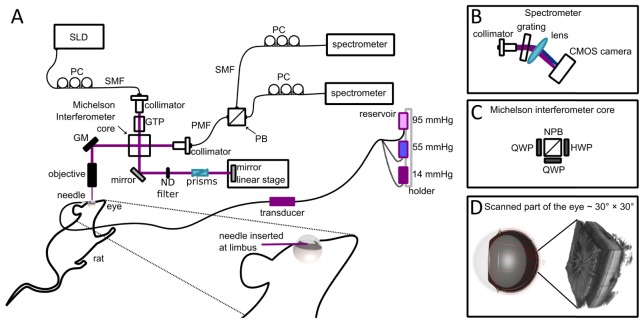
Sketch of the experimental setup. (A) Sketch of the OCT system and cannulation of the eye. SLD - superluminescent diode, PC - polarization controller, SMF - single mode fiber, PMF - polarization maintaining fiber, GM - galvanometer mirrors, QWP - quarter wave plate, HWP - half wave plate, NPB - non-polarizing beam splitter, GTP - Glan-Thomson polarizer, PB - polarizing beam splitter, ND filter - neutral density filter. (B) Sketch of the spectrometer used in the OCT system. (C) Core of the Michelson interferometer used in the system. (D) 3D rendering of a data set acquired during the experiment (field of view 30° × 30°).

For this study, a spectral domain PS-OCT with high axial resolution was used, as depicted in Fig. 1

. The system is described in detail elsewhere [25], here we provide just a brief description. The central wavelength of the system was 840 nm, the spectral bandwidth was 100 nm and the A-scan rate was 83 kHz. Axial resolution in tissue (n = 1.35) was measured as ~3.8 µm. All the cross-sectional images (B-scans), if not stated differently, were calculated as an average of 15 frames (5 frames repeated at one position, three consecutive positions separated by a distance of ~4 µm) for speckle reduction and signal to noise ratio improvement. The field of view was 30° × 30° which corresponds to approximately 1.5 mm × 1.5 mm on the retina.

### 2.2 Animals

Five male Sprague Dawley rats (albino, age range: 9 – 10 weeks) were purchased from the Medical University of Vienna breeding facility, Himberg, Austria. The average weight of the animals was 366 ± 34 g. Animals were kept under controlled lighting conditions (12 hours dark, 12 hours light) with food and water ad libitum. For the experiments, the animals were anesthetized using midazolam (Dormicum, Roche, Austria GmbH, Vienna, Austria, 1.0 ml/kg body weight), medetomidine (Domitor, Bayer Austria GmbH, Vienna, Austria, 0.2 ml/kg body weight), fentanyl (Fentanyl, Hameln Pharma Plus GmbH, Hameln, Germany, 0.3 ml/kg body weight) and ketamine (Ketasol; aniMedica GmbH, Senden, Germany, 0.1 ml/kg body weight). Eye drops (Oculotect, Novartis Pharma; Vienna, Austria) were applied to the rodent cornea during the measurements. Tropicamide (Mydriaticum; Agepha Pharmaceuticals, Vienna, Austria; topical) and phenylephrine (2.5%, topical) were used for pupil dilation. The animals were sacrificed by an overdose of sodium pentobarbital (Release, Richter Pharma AG, Wels, Austria) at the endpoint of the experiment. All experiments were performed in accordance with the Association for Research in Vision and Ophthalmology Statement for the Use of Animals in Ophthalmic and Vision Research and were approved by the ethics committee of the Medical University of Vienna and the Austrian Federal Ministry for Science and Research (protocol number GZ 66.009/0005-WF/V/3b/2016).

### 2.3 IOP experiment

In order to ensure the integrity of the eyes to be investigated, anesthetized animals were first imaged at their physiologic IOP prior to cannulation. Subsequently, the IOP was elevated in the right eye by cannulation of the anterior chamber with a needle (31 G) connected to a reservoir filled with 0.9% NaCl solution. A pressure transducer was connected between the needle and the reservoir as is illustrated in Fig. 1(A). In order to elevate the IOP, the reservoir was placed at different heights (ranging from 20 cm to 140 cm in respect to the eye) with increments of 5 mmHg (until 45 mmHg) and 10 mmHg (after 45 mmHg). The IOP level was hereby altered in a range of 14 to 105 mmHg and determined by the transducer connected between the reservoir and the rat eye. Denser sampling for the range until 45 mmHg was chosen since this range is usually used in longitudinal studies [27–30]. The time interval between the measurements at different IOP levels was approximately 4 minutes. Two additional measurements were performed 4 and 14 minutes after the IOP was lowered back to 14 mmHg at the end of the experiment.

### 2.4 Depression of the ONH

The internal limiting membrane (ILM) was automatically segmented in all data sets and its position was aligned according to the initial data set at 14 mmHg. Then, the difference between the average position of the ILM in an annulus (300 µm inner diameter, 700 µm outer diameter) centered at the ONH was calculated between the first measurement and successive measurements (i.e. 14 mmHg – 20 mmHg, 14 mmHg – 25 mmHg, and so on). The size of the annulus was chosen such that it contained only a confined peripapillary area, where the depression was present for every data set. Finally, the average of these values over 5 animals at each pressure was calculated.

### 2.5 Reflectivity changes of the RNFL

**Fig. 2:**
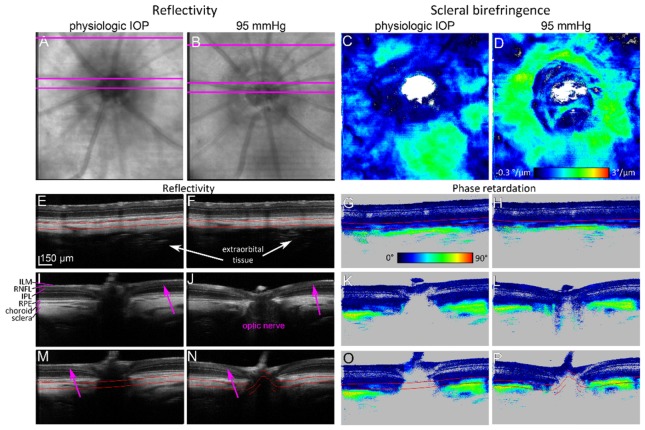
Exemplary high resolution PS-OCT images recorded during the experiment. (A) En face reflectivity projection at physiologic IOP, without the cannulation of the eye. (B) En face reflectivity projection at the pressure of 95 mmHg. A change of the intensity can be observed. (C) En face birefringence of the sclera at physiologic IOP and (D) at increased IOP of 95 mmHg. (E-P) Reflectivity and corresponding phase retardation B-scans recorded at the respective IOP levels. The red lines indicate the part of the sclera that was used in the birefringence evaluation. In (J) and (L), the optic nerve canal and optic nerve are visible. Low contrast of RNFL reflectivity is indicated by pink arrows. Extraorbital tissue is indicated by white arrows. ILM – internal limiting membrane, RNFL – retinal nerve fiber layer, IPL – inner plexiform layer, RPE – retinal pigment epithelium.

Relative reflectivity changes in the RNFL were evaluated as a ratio of the linear intensity in the RNFL and the linear intensity in the posterior retina (RNFL border until retinal pigment epithelium - RPE) [31], henceforth denoted as relative RNFL/retina reflectivity. Since the segmentation of the posterior border of the RNFL was error-prone at high IOP levels (decrease of RNFL contrast in the reflectivity, see Fig. 2(I), 2(J), 2(M), 2(N)

pink arrows), the ratio was calculated between a slab of tissue starting at the ILM with a thickness of 20 pixels (38 µm) and a slab of tissue starting 20 pixels (38 µm) posterior to the ILM and the RPE. The average reflectivity ratio was determined in an annulus with 550 µm inner diameter and 870 µm outer diameter centered at the ONH. The size of the annulus was defined in a way that it did not contain the ONH and that it fit in every data set (small variations in the eye position led to a slight shift of the field of view between measurements).

### 2.6 Retardation of the RNFL

RNFL retardation was calculated as described earlier [25] and thus is just described briefly as follows. In averaged reflectivity images, the ILM was segmented and its polarization state was used for corneal birefringence compensation [32]. After that, the segmented ILM position was moved by 20 pixels (38 µm) in posterior direction and average phase retardation was calculated in the slab 75 pixels (143 µm) below the segmentation line. Only pixels above an empirically chosen threshold (3 dB above the average noise level) were used for the calculation. Some data sets were excluded from the analysis, since not enough pixels met this criterion. Average retardation was then calculated in an annulus (550 µm inner diameter, 870 µm outer diameter). Since with increasing pressure, the reflectivity of the RNFL with respect to the retinal layers was changing (low contrast to adjacent layers, see Fig. 2(I), 2(J), 2(M), 2(N), pink arrows), it was not possible to reliably evaluate neither thickness nor birefringence of the RNFL.

### 2.7 Birefringence of the sclera

Scleral retardation was evaluated in a slab with an upper border 20 pixels (38 µm) posterior to the RPE (thereby excluding the choroid) and a thickness of 40 pixels (76 µm) indicated by red lines in Fig. 2. The thickness of the slab did not include the full depth of the sclera, but rather its anterior half. This was because the unambiguous range for retardation measurements is restricted to 0° - 90° due to the algorithm used [33]. Since the sclera is strongly birefringent, retardation values may exceed 90° at deeper locations and may then be wrapped below 90° again, see Fig. 2(O). In order to avoid such regions in the cumulative retardation measurements, only the aforesaid shallow slab of 40 pixels was used for birefringence calculations. For scleral birefringence measurements, corneal birefringence was compensated as for the RNFL retardation measurements described in section 2.6 (no additional RNFL birefringence compensation was performed). Similar to previous investigations, we assumed that retardation was linearly increasing with depth in the sclera and hence effective birefringence was calculated as the slope of a linear fit of phase retardation along each A-scan in this slab [34]. The birefringence values from all A-scans in the 3D data set were then displayed as en face birefringence map, see Fig. 2(C), 2(D). The average birefringence was finally calculated in the same annulus as described above (550 µm inner diameter, 870 µm outer diameter).

### 2.8 Ex vivo µCT imaging

Ex vivo µCT imaging was performed using a microXCT-400 (Carl Zeiss X-ray Microscopy, Pleasanton, CA) and a 0.4 × lens. The complete separated rat head was fixed in paraformaldehyde for 72 hours and washed in distilled water. To assess skull morphology, the unstained sample was scanned at 40 kV source voltage and 200 µA intensity. Projections were recorded at 3 s exposure time (camera binning = 4) and an angular increment of 0.25°. Reconstructed slices measured 512 × 512 pixel. Isotropic voxel resolution of reconstructed volumes was ~68 µm.

To assess morphology of soft tissues, the specimen was cut along the median sagittal plane using a band saw. The left half of the head was dehydrated in absolute ethanol and stained using 1% elemental iodine in absolute ethanol [35] for 14 days. Before scanning, the specimen was washed in absolute ethanol for 24 hours to remove unbound iodine from the tissues. The specimen was scanned at 130 kV source voltage and 60 µA intensity. Projections were recorded at 5 s exposure time (camera binning = 2) and an angular increment of 0.17°. Reconstructed slices measured 1024 × 1024 pixel. Isotropic voxel resolution of reconstructed volume was 18 µm.

The two µCT image stacks were exported in DICOM format and imported into Amira 6.1 (FEI Visualization Sciences Group, Mérignac Cédex, France) where they were co-registered based on normalized mutual information using the RegisterImages tool. Image segmentation of external eye muscles, the optic nerve, and the optic nerve sheath was done using manual segmentation tools. Based on image segmentation, models were created using the GenerateSurface tool and visualized by combined volume rendering of the skull (based on the µCT scan of the unstained specimen) and surface rendering of muscle models.

### 2.9 Statistical evaluation

To evaluate the correlation between measured parameters and the IOP, Spearman's correlation coefficient ρ was used which is applicable for monotonic relations. If the absolute value of ρ was in the range 1.00 – 0.70 it was considered as strong correlation, in the range 0.70 – 0.50 as moderate correlation and in the range 0.50 – 0.30 as weak correlation, respectively [36]. To evaluate statistical difference between superior and inferior halves of the sclera, Wilcoxon rank sum test was used (Statistics and Machine Learning toolbox in Matlab R2014b). P-values < 0.05 were considered statistically significant.

## 3. Results

### 3.1 Cupping of the ONH

**Fig. 3:**
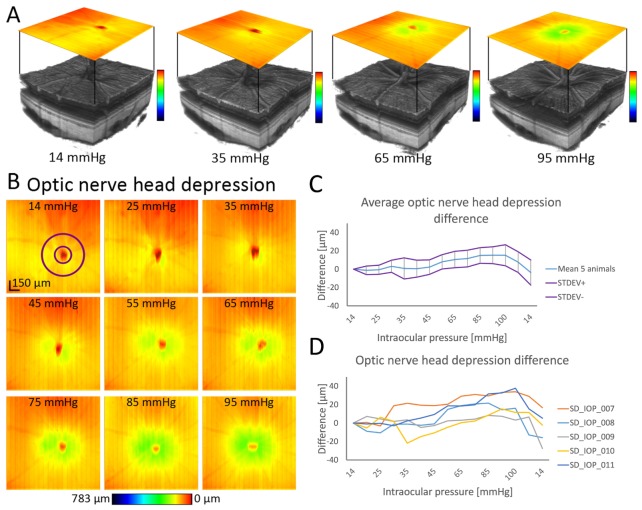
ONH depression (cupping). (A) 3D visualization of representative data sets together with en face depression maps. The location of the color bars at the right of each volume rendering indicates the color-coded displacement used in the depression maps. (B) En face depression maps for one animal aligned to the initial measurement with axial motion between B-scans corrected. (C) Average ONH depression measured in the annulus depicted in the first map in (B) in 5 animals (difference between data set at 14 mmHg and the successive data sets, mean ± standard deviation) (D) Individual ONH depression difference for each animal. Change in the depression is more apparent after 45 mmHg.

Cupping of the ONH is depicted in Fig. 3

in 3D fashion together with en face depression maps and the average depression difference (difference of the ILM position between baseline and set pressure after the alignment for axial motions) plotted against IOP for 5 animals. Exemplary depression maps for one animal are shown in Fig. 3(B) as a response to an increase in IOP from 14 to 95 mmHg. An apparent ONH extrusion is visible as a color change from yellow to green for pressures higher than 45 mmHg. The average depression difference evaluated in a circular annulus around the ONH for all evaluated animals is shown in the graph of Fig. 3(C) and individual values in Fig. 3(D). The average depression difference, in relation to the initial measurement at 14 mmHg, at the highest IOP of 105 mmHg was 15 µm ± 11 µm. After setting the IOP back to 14 mmHg, ONH cupping was diminished already after 4 minutes and was even more reduced in the second measurement at 14 mmHg after 10 additional minutes.

### 3.2 Identification of the scleral sling at severely elevated IOP

As the IOP increased, the reflectivity of retinal layers was changing. RNFL intensity decreased such that sometimes it was not possible to distinguish between RNFL and inner plexiform layer (IPL) due to a reduced contrast, see Fig. 2(J), 2(N).

**Fig. 4:**
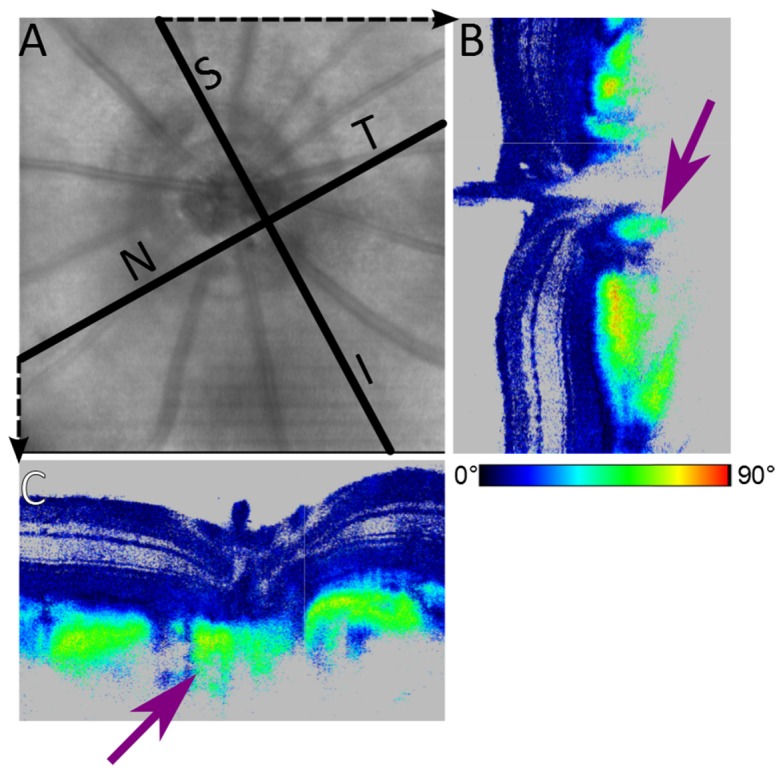
Scleral sling visualized at IOP ~95 mmHg. (A) En face reflectivity projection with marked position of cross sectional images. (B) Cross sectional phase retardation image in superior (S) – inferior (I) direction. (C) Cross sectional phase retardation image in nasal (N) - temporal (T) direction. Violet arrows point to the sclera between optic nerve and central retinal vessels (scleral sling).

Under physiologic IOP, the retinal and scleral parts of the ONH were not visible very well due to shadowing by the retinal vessels. However, when IOP increased, blood perfusion was reduced and vessel diameters decreased. Thus less blood blocked the light beam which resulted in improved visibility of the scleral part of the ONH, see Fig. 2(I)-2(J). Also, the scleral rim around the ONH became more visible, and we were able to distinguish the sclera between the central retinal artery and optic nerve, see Fig. 4(C)

. The so-called scleral sling, which was previously reported in a histological study [20], can clearly be identified in the PS-OCT phase retardation images.

### 3.3 Changes of the scleral birefringence

**Fig. 5:**
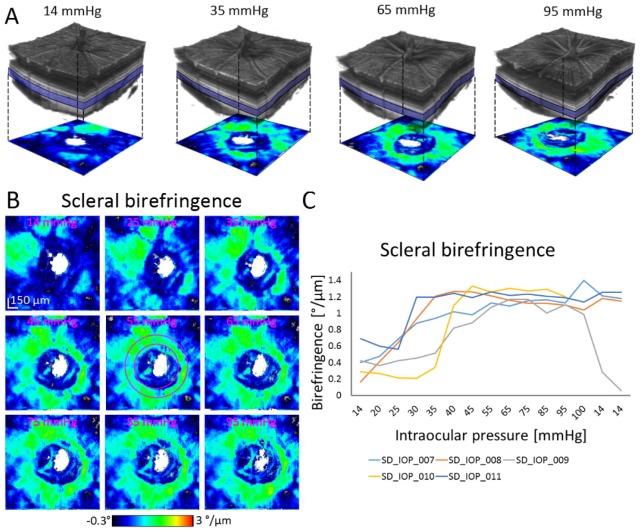
Scleral birefringence. (A) 3D visualization of an exemplary reflectivity data set. Scleral birefringence was evaluated in the depicted bluish slab and is shown as a birefringence en face map beneath the volume rendering. (B) En face maps of scleral birefringence for an exemplary animal as response to elevated IOP. (C) Average scleral birefringence measured in the annulus depicted in the middle map in (B) in 5 animals. Scleral birefringence increased with elevated IOP. After lowering back to 14 mmHg, restored scleral birefringence values were observed in just one animal.

**Fig. 6:**
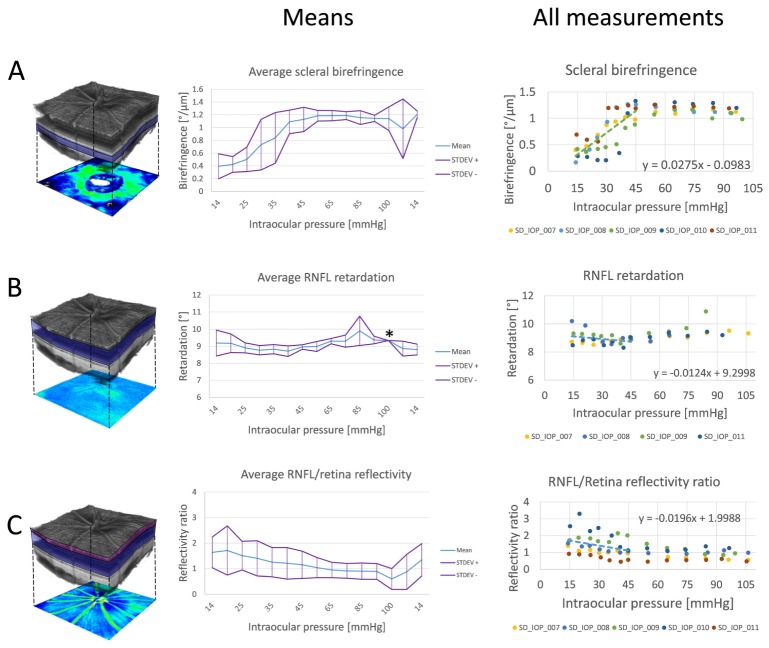
Retinal and scleral changes as a response to IOP elevation. (A) Average scleral birefringence shows an increase as a response to an elevated IOP from 14 to 45 mmHg. (B) The RNFL retardation does not show any strong correlation with IOP variation. (C) The RNFL/retina reflectivity ratio vs. IOP shows a moderate, negative correlation. In the middle column, the mean quantities ( ± standard deviation - STDEV) are plotted. All data points are plotted in the right column, where each dot represents the respective quantity for one animal at a given IOP. Measurements after lowering the IOP back to 14 mmHg are excluded from these plots. Linear regressions are shown for the lower IOP range of 14 – 45 mmHg. Value marked by asterisk was retrieved just from one data set.

Exemplary phase retardation B-scans are depicted in Fig. 2(G), 2(H), 2(K), 2(L), 2(O), 2(P). From such phase retardation images, scleral birefringence was calculated as described in section 2.7. Figure 5(A)

shows rendered reflectivity data sets at various IOP levels together with scleral birefringence maps. Figure 5(B) shows exemplary scleral birefringence maps as a response to IOP changes from 14 to 95 mmHg and the annulus used for the calculation of average circumpapillary scleral birefringence. The average circumpapillary birefringence values from 5 animals are plotted in Fig. 5(C). Scleral birefringence was increasing as IOP was elevated and reached a plateau like phase at ~45 mmHg, see Fig. 6(A)

, where an average birefringence of 1.16 ± 0.04 °/µm was observed. After lowering IOP back to 14 mmHg, scleral birefringence did not promptly return to the initial values, not even at the second measurement after 10 additional minutes, except for one data set. This data set suffered from decreased image quality due to ocular deformations induced by the experiment for the last two measurements which could have affected quantitative birefringence measurements. In Fig. 6(A), scatter plots of all measured values (except for the final two measurements taken after reducing IOP back to 14 mmHg again) are shown together with plots of measurements where the pressure was lowered back to 14 mmHg. Each colored dot represents the birefringence value retrieved from one 3D data set. Scleral birefringence exhibits a moderate positive correlation with IOP (ρ = 0.66; p = 2.82 × 10^−9^). Scleral birefringence also showed a positive correlation in the range of 14 – 45 mmHg (ρ = 0.75; p = 2.33 × 10^−7^). This IOP range was investigated separately since it has been frequently used in longitudinal glaucoma model experiments [27–30]. The average birefringence increase in this range was 2.75 × 10^−2^ °/µm/mmHg, corresponding to a dimensionless birefringence increase of 6.42 × 10^−5^ mmHg^−1^ at 840 nm wavelength. There was no significant difference in birefringence between the superior and inferior halves of the annulus (Wilcoxon rank sum test, p = 0.4418).

### 3.4 Changes of the RNFL retardation and reflectivity

The RNFL retardation was evaluated in a slab of the posterior retina as depicted in Fig. 6(B). Retardation changes as a response to IOP elevation are shown as the mean for 4 animals as well as in the scatter plot of Fig. 6(B). The correlation analysis revealed a weak correlation between RNFL retardation and IOP (ρ = 0.39; p = 7.48 × 10^−3^). In the range of 14 – 45 mmHg, we did not find any statistically significant correlation (ρ = −0.16, p = 0.42).

In contrast, the reflectivity of the RNFL was changing substantially when IOP was elevated, see Fig. 2. The relative RNFL/retina reflectivity was evaluated as depicted in Fig. 6(C) where the red slab corresponds to the RNFL region and the blueish slab to the posterior retina region. The reflectivity ratio showed a moderate negative correlation with IOP (ρ = −0.51; p = 1.79 × 10^−5^), see Fig. 6(C). No statistically significant correlation was found in the range of 14 – 45 mmHg, (ρ = −0.32; p = 0.06). The quantitative parameters as a response to IOP elevation are summarized in Table 1

**Table 1:** Correlation coefficients ρ for scleral birefringence, RNFL retardation and relative RNFL/retina reflectivity vs. IOP together with slopes of the respective linear regression lines. Correlation coefficients were used to analyze monotonic relations. In addition, linear regression analyses were performed.

			14 - 105 mmHg					14 - 45 mmHg		
			
			Linear regression	Spearman ρ			Linear regression		Spearman ρ	

			slope	ρ	p-value		slope		ρ	p-value
Scleral birefringence			N/A	0.66	2.82 × 10^−9^			2.75 × 10^−2^ °/µm/mmHg	0.75	2.33 × 10^−7^
RNFL retardation			7.4 × 10^−3^ °/mmHg	0.39	7.48 × 10^−3^			−1.24 × 10^−2^ °/mmHg	−0.16	0.42
RNFL/retina reflectivity			−1.0 × 10^−2^ mmHg^−1^	−0.51	1.79 × 10^−5^			−1.96 × 10^−2^ mmHg^−1^	−0.32	0.06

.

### 3.5 Ex vivo µCT imaging of the rat eye and head

Due to the absence of pigmentation in the Sprague Dawley rat, OCT can deeply penetrate into the posterior parts of the rat eye and thus the sclera was nicely visualized in all experiments. Sometimes, the deep penetration also revealed structures even beyond the sclera (see, for instance, Fig. 2(F)). To investigate the anatomical structure and validate the scleral structure visualized by PS-OCT in vivo, ex-vivo µCT imaging was performed for one rat after the endpoint of the experiment.

**Fig. 7:**
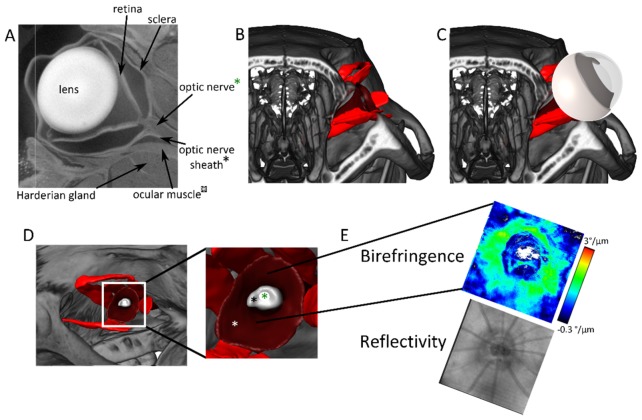
Ex vivo µCT scan of a rat eye and head. (A) Tomogram through the rat eye. Densely packed structures like Harderian gland and muscles are visible. Deformations of the eye are due to preparation artifacts of the sample. (B) Rendered µCT data from the rat head. (C) Rendered µCT data together with sketch of the eye. (D) µCT visualization from the “en face” view similar as used for OCT scans. Color code: Red: oblique and rectus muscles; dark red: retractor bulbi, marked with white asterisk; grey: optic nerve sheath, marked with black asterisk; white: optic nerve, marked with green asterisk. (E) Birefringence and reflectivity en face projections of OCT data corresponding to the location in (D).

A transverse slice through the rat eye imaged using µCT is shown in Fig. 7(A)

. Posterior to the eye, orbital muscles and the Harderian gland are visible. The nerve sheath which is surrounding the optic nerve is noticeable. These structures are in direct contact with the outer sclera. A 3D rendering of the rat skull is shown in Fig. 7(B)-7(D). The orbital muscles are segmented (red; dark red) together with the optic nerve (white) as well as the nerve sheath (grey). The interconnection of nerve sheath and sclera corresponds to the location of high scleral birefringence as shown in Fig. 7(E).

## 4. Discussion

In this article, a rat model of short term IOP elevation was investigated using high resolution PS-OCT. This imaging method was demonstrated by our group to be capable of investigating the peripapillary sclera of healthy albino rats [34] and the retina of healthy albino and pigmented rats and mice [25] in the past. There, it was shown that PS-OCT can access birefringence of sclera and RNFL in rodents.

Ex vivo measurements of scleral birefringence and in vivo measurements of various parameters in rat models of increased IOP were performed by several groups previously. Reflectivity and thickness changes of retinal layers (such as the ganglion cell layer) [24,37,38] were reported as well as perfusion changes [19] and functional changes [39]. With this article, we strived to extend the knowledge with in vivo measurements of polarization properties of posterior eye structures at elevated IOP.

In this study, we performed a quantitative evaluation of the ONH depression as a difference between average ONH position in the measurement at 14 mmHg and of elevated IOP up to 105 mmHg. When plotted against the IOP, above ~45 mmHg the change is more apparent, depression rises and finally goes back when the IOP is lowered to 14 mmHg again, see Fig. 3. Below 45 mmHg, the change oscillates around zero, which can be accounted to the small imperfections in co-registration of the data sets. Detectable displacement of the ONH surface was also noticed by Fortune et al. [40] and recently published in a detailed study of neural rim tissue compression in nonhuman primates [41]. In addition, the result of the latter study showed that the thinning of the ONH neuroretinal rim is more pronounced than the thinning of the peripapillary RNFL tissue. In another study in rodents [21], where retinal thickness around ONH was evaluated at IOP 15 mmHg, 50 mmHg and 70 mmHg, decreased retinal thickness in the area around Bruch’s membrane opening was reported. However, displacement of the ONH itself was not quantified. Our findings are in strong agreement with results of Zhi et al. [18]. They described ONH contour change qualitatively at various IOP levels (10 mmHg – 100 mmHg) in rats and reported that contour change was not apparent until ~50 mmHg. Above 60 mmHg, they reported retinal compression, distortion of retina and choroid and compaction of the ONH. Choh et al. [39] quantitatively evaluated Bruch’s membrane opening displacement (similar method used as in reference [18]) at IOP elevated to 35 mmHg in rats. They reported significant displacement even at this comparatively low pressure.

We investigated scleral birefringence as well as reflectivity changes and RNFL retardation simultaneously and evaluated their dependence on IOP. RNFL reflectance was observed to change before its thickness [42], and the most stable way to assess this change reliably was reported to be the ratio of RNFL/retina posterior to the RNFL [31]. Therefore, we used this parameter for the evaluation of reflectivity changes. We found that this parameter had moderate (negative) correlation with IOP (ρ = −0.51; p = 1.79 × 10^−5^). This correlation is in agreement with previous findings, where it was shown that reflectance of the RNFL has a strong directional nature [43].

Since birefringence is connected to the biomechanical properties of the sclera [11], the changes during the experiment most likely reflect changes in structural stiffness. Scleral birefringence showed moderate, positive correlation (ρ = 0.66; p = 2.82 × 10^−9^). The positive correlation of the scleral birefringence with increasing IOP differs from the finding reported by Yamanari et al. [3]. They observed a moderate negative correlation (Pearson's correlation coefficient = −0.63; p = 0.002) in the anterior segment of human sclera in vivo and no significant correlation in an ex vivo experiment with porcine eyes [3]. This difference between their results and those reported here could have several reasons. The mentioned study was performed on human eyes in vivo (physiologic range of IOP) and on ex vivo porcine eyes (controlled IOP). As such, birefringence was investigated in the anterior segment of the sclera. In contrast, in the study presented here, the posterior part of the sclera was investigated in vivo in rats. The scleral fiber alignment, which is responsible for the birefringence, in rat eyes depends on location [44], with a higher degree of orientation at the limbus than in peripapillary area. Around the ONH, the preferential orientation of the fibers is circular with its birefringence depending on the eccentricity [34]. In the anterior segment of the sclera, there is no surrounding structure acting against the expansion of the sclera when the pressure is increased. The expansion of the eye wall would account for the birefringence decrease, since the highly organized collagen fibers in the anterior eye are not only compressed in z direction, but also spread in the x-y plane. As the posterior sclera is less organized than its anterior part, tissue compression could result in higher organization of the fibers which would result in a birefringence increase. Also, posterior to the eye, structures such as muscles, Harderian gland, fatty tissue and optic nerve are densely packed (see Fig. 7) and therefore stiffness and compressibility of the surrounding are likely to influence the expansion of the eye. In an ex vivo µCT image of a rat head in Fig. 7, the relation between eye ball, ocular muscles and optic nerve is visualized. An interconnection of the optic nerve sheath with sclera can be identified in the posterior peripapillary region, see Fig. 7(D). Yamanari et al. [3] used a different wavelength in their experiment (1310 nm compared to 840 nm used in this study). But since the ultrastructure creating the form birefringence is much smaller than the used wavelengths, the outcome should not differ qualitatively. Therefore, the difference in our and their results should not be due to different wavelengths. It would be useful to investigate also the functional state of the retina [39] together with scleral birefringence in order to find out whether scleral birefringence correlates with functional damage of the retina and in particular of the ONH. Threshold for functional damage is around 30 mmHg [45].

Findings that the optic nerve is damaged focally in the superior area [46], that there is a greater loss of axons in superior retina [47] and that retinal thickness is reduced in the superior quadrant [21] in rats were published in the past. Morrison et al. [48] suggested that the reason for these pathological alterations could be the central retinal vessels entering inferior to the optic nerve. This way, only the superior part of the ONH is in contact with sclera and scleral stress can be transmitted to the optic nerve. We did, however, not find any significant difference in birefringence between the superior and inferior half (Wilcoxon rank sum test, p = 0.4418).

A weak correlation was found between RNFL retardation and IOP (ρ = 0.39, p = 7.48 × 10^−3^), although the range of measured values (minimal value 8.3°, maximal value 9.5°) was rather small compared to the standard deviation inside the annulus volume of 5°. Since RNFL thickness could not be accessed reliably at increased IOP, it was not possible to tell if an IOP increase was accompanied by RNFL birefringence changes. Also, RNFL retardation values could be influenced by noise, since the values could not be thresholded according to the RNFL thickness as in our earlier work [25]. In that work, only retardation values in areas of RNFL thickness above 26 µm were mapped. It was, however, reported that RNFL retardation is changed in a non-human model of glaucoma [49,50] and in glaucoma patients [51–55]. This change was believed to be due to the progressive loss of retinal nerve fibers and/or changes in the injured axons. The short-term nature of our experiment was probably not sufficient to produce enough damage to the retinal nerve fibers even at highest IOP levels.

In addition to the correlation of scleral birefringence, RNFL retardation and relative RNFL/retina reflectivity with IOP over the entire range of data points spanning from 14 to 105 mmHg, we separately investigated the correlation for the IOP range from 14 mmHg to 45 mmHg. In this range, scleral birefringence showed a stronger correlation with IOP than for the whole range of measurements. This suggests that the sclera does not produce a gradual change under increasing IOP, but the response rather has two phases: First a dynamic phase, where the birefringence changes rapidly with IOP and after that, a less dynamic, or plateau like phase, where birefringence stays rather constant or changes slowly. This is also visible in Fig. 6(A) and suggests that at IOP ~45 mmHg, the sclera reached the point where the biomechanical response to the increased IOP is slower or even stable and resembling the stress-strain hysteresis reported by Curtin [56]. The fact that scleral birefringence did not return to its baseline value after lowering the IOP back to 14 mmHg could mean that our waiting time was not sufficient for scleral recovery or even that the scleral microstructure might have been irreversibly damaged. To test this hypothesis, it would be interesting to investigate the scleral microstructure ex vivo in normal rats and in rats that have undergone acute IOP increase. RNFL retardation and relative RNFL/retina reflectivity exhibited no statistically significant correlation with IOP in this lower range of IOP values.

In summary, the polarization changes observed in the PS-OCT imaging study presented in this work suggest that birefringence of the sclera may be a promising IOP-related parameter to investigate. As a next step, an animal study will investigate the effect of longitudinally increased IOP on the polarization properties of the retina and sclera. Finally, the results from this preclinical study may provide a basis for defining PS-OCT parameters for clinical imaging studies focusing on the peripapillary sclera in human glaucoma patients.

## 5. Conclusion

In this article, PS-OCT was used to study the impact of IOP on scleral birefringence, RNFL retardation and reflectivity changes in the retina. Both scleral birefringence and relative RNFL/retina reflectivity showed a similar correlation with IOP for the range of 14 mmHg – 105 mmHg. RNFL retardation exhibited only a weak correlation with IOP in the same range. In the IOP range of 14 mmHg – 45 mmHg, RNFL retardation and relative RNFL/retina reflectivity did not exhibit a statistically significant correlation, whereas scleral birefringence showed moderate correlation with IOP. In contrast to the rather monotonous change of ONH cupping and the RNFL/retina reflectivity ratio, the response of scleral birefringence to the IOP increase could be split into two phases: a dynamic phase and a plateau-like phase.

## Disclosures

None.
